# Major heart defects: the diagnostic evaluations of first-year-olds

**DOI:** 10.1186/s12887-021-02997-2

**Published:** 2021-11-30

**Authors:** Jan Pavlicek, Eva Klaskova, Sabina Kapralova, Alzbeta Moravova Palatova, Alicja Piegzova, Richard Spacek, Tomas Gruszka

**Affiliations:** 1 Department of Pediatrics, University Hospital Ostrava and Faculty of Medicine, Ostrava University, Ostrava, Czech Republic; 2 Biomedical Research Center, University Hospital Hradec Kralove, Hradec Kralove, Czech Republic; 3 Department of Pediatrics, Palacky University Hospital, Palacky University, Olomouc, Czech Republic; 4 Department of Obstetrics and Gynaecology, University Hospital Ostrava, Ostrava, Czech Republic

**Keywords:** Congenital heart defect, Cyanosis, Murmur, Symptom

## Abstract

**Background:**

Severe or critical congenital heart defects (CHDs) constitute one third of the heart defect cases detected only after birth. These prenatally unrecognised defects usually manifest as cyanotic or acyanotic lesions and are diagnosed postnatally at various times. The aim of the study was to identify their clinical symptoms and determine individual risk periods for CHD manifestation.

**Methods:**

Data were assessed retrospectively based on a cohort of patients born between 2009 and 2018 in a population of 175,153 live births. Occurrence of the first symptoms of CHD was classified into: early neonatal (0–7 days), late neonatal (8–28 days), early infancy (1–6 months), or late infancy (6–12 months). The first symptom for which the child was referred to a paediatric cardiologist was defined as a symptom of CHD.

**Results:**

There were 598 major CHDs diagnosed in the studied region, 91% of which were isolated anomalies. A concomitant genetic disorder was diagnosed in 6% of the cases, while 3% presented extracardiac pathology with a normal karyotype. In total, 47% (282/598) of all CHDs were not identified prenatally. Of these, 74% (210/282) were diagnosed as early neonates, 16% (44/282) as late neonates, and 10% (28/282) as infants. The most common symptoms leading to the diagnosis of CHD were heart murmur (51%, 145/282) and cyanosis (26%, 73/282). Diagnosis after discharge from the hospital occurred in 12% (72/598) of all major CHDs. Ventricular septal defect and coarctation of the aorta constituted the majority of delayed diagnoses.

**Conclusions:**

In conclusion, murmur and cyanosis are the most common manifestations of prenatally undetected CHDs. Although most children with major CHDs are diagnosed as neonates, some patients are still discharged from the maternity hospital with an unidentified defect.

**Supplementary Information:**

The online version contains supplementary material available at 10.1186/s12887-021-02997-2.

## Background

Congenital heart defects (CHDs) are the most frequently observed congenital defects in the human population, representing up to 40% of all congenital malformations [1, 2]. The incidence of CHD varies from 4 to 50 cases per 1000 live births, but when minor ventricular septal defects are also included, the incidence rises to 75 cases per 1000 live births [3]. The aetiology of CHDs is complex, with genetic and environmental factors known to play a part [3]. Indeed, most defects likely have an underlying multifactorial aetiology [4, 5]. Approximately 20–25% of CHDs have a precise genetic cause [4, 6]. Most CHDs can be prenatally detected, and the overall success of prenatal CHD diagnoses has generally improved. Foetal echocardiography displays high sensitivity and specificity for the detection of major CHDs [7, 8]. However, some children are born with prenatally undetected CHDs that are diagnosed only after birth. Of the total number of CHDs, 35% are considered severe or critical, and these defects can be health- and life-threatening. Therefore, it is crucial to diagnose these defects as soon as possible. Symptoms of heart disease may develop slowly or very rapidly and worsen in the maternity hospital or at home. In critical and severe CHDs, the most common symptom is the development of hypoxemia and heart failure. In other defects, the most common symptoms are pulmonary symptomatology, failure to thrive, or murmur. CHDs have an incidence of 2–75 per 1000 live births [1, 3, 6, 9, 10]. Moreover, although the association between the first CHD manifestations and the different stages of childhood is less frequently studied, this information is very important for paediatricians.

Thus, the aim of this work was to identify clinical symptoms and determine the individual risk periods for CHD manifestation.

## Methods

The data were assessed retrospectively based on a cohort of patients born between 2009 and 2018 in a population of 175,153 live births. This study was conducted at the Department of Paediatric and Prenatal Cardiology, University Hospital Ostrava, Czech Republic, and at the Department of Paediatrics, Palacky University Hospital, Olomouc, Czech Republic. These centres are tertiary referral centres for paediatric cardiology that offer prenatal care and care for critical and severe CHDs. They serve a population of about 1,830,000 inhabitants, with 18,200 live births per year.

The study included only children born with CHD. Cases of pregnancies terminated for CHD were not included in this study. All cases in the study underwent at least one ultrasound examination between the gestational age 20 and 22 weeks performed by a gynaecologist. When a cardiac defect was suspected, it was confirmed or excluded by a paediatric cardiologist. All prenatally detected cases were examined by a paediatric cardiologist in one of the two centres and, following a planned delivery, also re-examined there. Children with potentially critical prenatally detected CHDs were delivered in a surgical theatre to allow urgent treatment or surgery immediately; hence, not all the symptoms typical of CHD had time to manifest in such newborns. Therefore, the number of prenatally detected cases was recorded but they were not analyzed further. The aim was to analyse the symptoms of prenatally unidentified CHD. Children with prenatally unknown defects were either born in the mentioned centres or were transported there for hospitalization and examination from other catchment hospitals.

The echocardiograph ultrasound systems used were the GE Vivid 7 and Vivid E9 (GE Healthcare, Horten, Norway), and Voluson E8 and E10 (GE Healthcare, Zipf, Austria). These systems were equipped with multifrequency wide-bandwidth transducers: a C2–9 single crystal abdominal convex 2.3–8.4 MHz transducer, 6S phased-array 2.4–8.0 MHz transducer, 5S phased-array 3.0–9.0 MHz transducer, and M5Sc active matrix single crystal phased array 1.5–4.6 MHz transducer. The echocardiograph system provided basic 2D imaging, M-mode and anatomical M-mode, colour Doppler imaging, and pulse wave and continuous wave Doppler imaging.

Major CHD was defined as critical or severe heart defect which required an intervention (heart surgery or catheterisation procedure) during the first year of age [11]. The diagnosis of a single heart defect or large vessel was described, while the diagnoses of complex cardiac abnormalities were classified according to the dominant heart lesion. The atrioventricular septal defects constituted a primary diagnostic lesion for all complex heart lesions reported in the study. A double-outlet right ventricle diagnosis was made when more than 50% of the aorta or the pulmonary trunk overrode the ventricular septal defect. A single ventricle was classified as a univentricular atrioventricular connection with a double inlet or a common atrioventricular valve. When an atrioventricular connection was absent, the diagnosis of either tricuspid or mitral atresia was made. Hypoplastic left heart syndrome was defined as a heart with a small left ventricle and flow reversal in the aortic arch. Coarctation of the aorta with a ventricular septal defect was classified as coarctation of the aorta [12, 13]. Persistent ductus arteriosus is a specific category; as ductus arteriosus is a physiological part of the foetal circulation, it cannot be classified as prenatally unidentified. In the study, defects were included into this separate category only if this defect was isolated; where it was a part of a complex defect, it was classified according primary defect.

According to previous studies, we divided major CHDs into two groups: cyanotic and acyanotic lesions [3]. The following defects were included in cyanotic CHDs: interruption of the aortic arch, common arterial trunk, double outlet right ventricle, Ebstein’s anomaly, hypoplastic left heart syndrome, pulmonary atresia with ventricular septal defect, pulmonary atresia with intact ventricular septum, tetralogy of Fallot, total anomalous pulmonary venous return, transposition of the great arteries, tricuspid atresia, and single ventricle. The following defects were included in acyanotic CHDs: aortic stenosis (severe), atrioventricular septal defect, coarctation of aorta, corrected transposition of the great arteries, persistent ductus arteriosus (large), pulmonary stenosis (severe), ventricular septal defect (large).

The occurrence of the first symptoms of CHD was monitored in the following defined periods of childhood: early neonatal (0–7 days), late neonatal (8–28 days), early infancy (1–6 months), and late infancy (6–12 months). The first clinical manifestation for which the child was referred to a paediatric cardiologist was defined as the leading symptom of CHD.

The study was approved by the local Ethics Committee (Ethics Committee of the University Hospital and the Faculty of Medicine, Palacky University, Olomouc). The study involved only paediatric patients. Parental signed consent was obtained for all examinations and data processing.

### Statistical analysis

A descriptive analysis was used for the description of the patient group. Pearson’s chi-square test or Fisher’s exact test were used for categorical variables as appropriate. The level of significance α for the probability of a type-I error (*p* or *p* value) was set at 0.05 for all tests. Analyses were performed using STAT software (Stata version 14, StataCorp LP, College Station, TX, USA).

## Results

### Basic evaluation

A total of 598 severe heart defects were diagnosed in a study population of 175,153 live births occurring between 2009 and 2018. CHDs mostly (91%, 541/598) manifested as isolated anomalies. A concomitant genetic disorder was diagnosed in 6% (37/598) of the cases, and extracardiac pathology with a normal karyotype was present in 3% (20/598) of the cases. Associated abnormalities in the prenatal and postnatal groups, and genetic diseases are shown in the Supplement (Supplementary Tables 1 and 2).

In total, 47% (282/598) of all CHDs were not identified prenatally. The percentages of prenatally unidentified cases for the different CHDs were as follows: ventricular septal defect, 72% (72/100); atrioventricular septal defect, 29% (19/65); coarctation of the aorta, 58% (33/57); transposition of the great arteries, 44% (24/55); tetralogy of Fallot, 37% (20/54); persistent ductus arteriosus, 100% (46/46); hypoplastic left heart syndrome, 9% (4/45); pulmonary stenosis, 44% (17/39); double outlet right ventricle, 38% (14/37); aortic stenosis, 37% (12/32); pulmonary atresia with ventricular septal defect, 25% (3/12); common arterial trunk, 45% (5/11); Ebstein’s anomaly, 45% (5/11); tricuspid atresia, 20% (2/10); single ventricle, 12% (1/8); pulmonary atresia with intact ventricular septum, 0% (0/6); interruption of the aortic arch, 50% (2/4); corrected transposition of the great arteries, 0% (0/3); and total anomalous pulmonary venous return, 100% (3/3).

Among the newborns with prenatally undetected CHD, 52% (147/282) were males. The median age of the mothers having newborns diagnosed with CHDs was 29 (range 16–46). In total, 15% (42/282) of the mothers had a history of risk factors. The most common (38/282) maternal diseases were pregestational diabetes, hypertension, thyroid disease, rheumatic and autoimmune disease, oncological disease, and haematological and coagulation disorders. No intrauterine infection, including TORCH, was found in the history of the studied cases. In 4 cases, the mother herself was treated for moderate or severe CHD. Vaginal birth occurred in 63% (177/282) of the children born with CHDs, and there was no significant perinatal asphyxia, even in critical cases. Details of the perinatal period, and gender are given in Table 1. The most common CHD was a ventricular septal defect, and the least common was a single ventricle. The group of cyanotic defects included 83 cases, while the group of acyanotic defects included 199 cases.

**Table 1:** Prenatally undetected major heart defects, newborns data

Congenital heart defect (***n*** = 282)	week of delivery median (range)	vaginal delivery	Apgar score at 5 min below 7 points	weight in grams median (range)	male gender
		N (%)	N (%)		N (%)
**Cyanotic defects**					
Transposition of the great arteries (*n* = 24)	38 (33–42)	19 (79)	3 (13)	3000 (1770–4370)	16 (67)
Tetralogy of Fallot (*n* = 20)	38 (37–42)	14 (70)	2 (10)	3010 (1880–4050)	9 (45)
Double outlet right ventricle (*n* = 14)	39 (36–40)	10 (71)	1 (7)	2990 (1450–4470)	9 (64)
Common arterial trunk (*n* = 5)	36 (34–38)	4 (80)	1 (20)	2600 (1300–3050)	3 (60)
Ebstein’s anomaly (*n* = 5)	39 (38–41)	3 (60)	0	2900 (2680–3390)	3 (60)
Hypoplastic left heart syndrome (*n* = 4)	40 (37–41)	3 (75)	0	3550 (3180–4100)	3 (75)
Pulmonary atresia/ventricular septal defect (*n* = 3)	40 (33–40)	2 (67)	0	2930 (1740–2320)	1 (33)
Total anomalous pulmonary venous return (*n* = 3)	40 (39–40)	2 (67)	0	3650 (3130–4620)	2 (67)
Interrupted aortic arch (*n* = 2)	40 (39–41)	1 (50)	0	3700 (3500–3900)	1 (50)
Tricuspid atresia (*n* = 2)	37 (32–41)	2 (100)	0	2500 (1500–3500)	2 (100)
Single ventricle (*n* = 1)	35	0	0	1690	1 (100)
**Acyanotic defects**					
Ventricular septal defect (*n* = 72)	39 (30–42)	51 (71)	1 (1)	2800 (1180–4520)	34 (47)
Persistent ductus arteriosus (*n* = 46)	27 (24–40)	16 (35)	8 (17)	940 (540–4320)	20 (43)
Atrioventricular septal defect (*n* = 19)	39 (34–41)	11 (58)	3 (16)	3195 (1870–4100)	10 (53)
Pulmonary stenosis (*n* = 17)	39(35–42)	12 (71)	1 (6)	2880 (2300–3960)	8 (47)
Aortic stenosis (*n* = 12)	38 (35–40)	7 (58)	0	2800 (1900–3900)	7 (58)

### Time of diagnosis

A total of 74% (210/282) of the children with CHD were diagnosed as early neonates, usually during their stay at the maternity hospital (Table 2). In 100% of the cases, the following defects occurred at this early age: transposition of the great arteries, tetralogy of Fallot, common arterial trunk, interruption of the aortic arch, hypoplastic left heart syndrome, pulmonary and tricuspid atresia, and single ventricle. Coarctation of the aorta and Ebstein’s anomaly were the least frequent diagnoses in the earliest period, which in one third of the cases were manifested in the late neonatal period when the newborn was discharged from the maternity hospital. Diagnosis after discharge occurred in 26% (72/282) of the hospitalised prenatally undetected cases and in 12% (72/598) of all major CHDs. In infancy, the finding of ventricular septal defect, pulmonary stenosis, coarctation of the aorta, and Ebstein’s anomaly exceeded 10%, and these defects had a greater risk of late detection in infancy than other major CHDs (18% versus 3%, Pearson’s chi-square test, *p* < 0.001). All defects except isolated cases of ventricular septal defect and persistent ductus arteriosus were diagnosed by 6 months of age.

**Table 2:** Time of diagnosis of major heart defects in the neonatal and infant periods

Congenital heart defect (***n*** = 282)	Newborn	Newborn	Infant	Infant
	0–7 days	8–28 days	1–6 months	7–12 months
	N (%)	N (%)	N (%)	N (%)
**Cyanotic defects**				
Transposition of the great arteries (*n* = 24)	24 (100)	0 (0)	0 (0)	0 (0)
Tetralogy of Fallot (*n* = 20)	20 (100)	0 (0)	0 (0)	0 (0)
Double outlet right ventricle (*n* = 14)	13 (93)	0 (0)	1 (7)	0 (0)
Common arterial trunk (*n* = 5)	5 (100)	0 (0)	0 (0)	0 (0)
Ebstein’s anomaly (*n* = 5)	2 (40)	2 (40)	1 (20)	0 (0)
Hypoplastic left heart syndrome (*n* = 4)	4 (100)	0 (0)	0 (0)	0 (0)
Pulmonary atresia/ventricular septal defect (*n* = 3)	3 (100)	0 (0)	0 (0)	0 (0)
Total anomalous pulmonary venous return (*n* = 3)	3 (100)	0 (0)	0 (0)	0 (0)
Interrupted aortic arch (*n* = 2)	2 (100)	0 (0)	0 (0)	0 (0)
Tricuspid atresia (*n* = 2)	2 (100)	0 (0)	0 (0)	0 (0)
Single ventricle (*n* = 1)	1 (100)	0 (0)	0 (0)	0 (0)
**Acyanotic defects**				
Ventricular septal defect (*n* = 72)	50 (69)	7 (10)	13 (18)	2 (3)
Persistent ductus arteriosus (*n* = 46)	29 (63)	14 (31)	2 (4)	1 (2)
Coarctation of the aorta (*n* = 33)	15 (46)	13 (39)	5 (15)	0 (0)
Atrioventricular septal defect (*n* = 19)	15 (79)	3 (16)	1 (5)	0 (0)
Pulmonary stenosis (*n* = 17)	12 (70)	3 (18)	2 (12)	0 (0)
Aortic stenosis (*n* = 12)	10 (83)	2 (17)	0 (0)	0 (0)

As expected, when newborns were divided into two groups based on the presence of cyanotic and acyanotic defects, those with cyanotic defects had earlier manifestations [early neonatal: cyanotic 95% (79/83) vs. acyanotic 66% (131/199), Fisher’s exact test, *p* < 0.001; late neonatal: cyanotic 2% (2/83) vs. acyanotic 21% (42/199), Fisher’s exact test, *p* < 0.001; early infancy: 2% (2/83) vs 12% (23/199), Fisher’s exact test, *p* = 0.011; and late infancy: 0% vs 2% (3/199), Fisher’s exact test, *p* = 0.56, respectively].

### Symptoms leading to diagnosis

The most common symptoms leading to the diagnosis of CHD were heart murmur (51%, 145/282) and cyanosis (26%, 73/282) (Table 3). Cyanosis was the most common symptom in the group of cyanotic defects including transposition of the great arteries, tetralogy of Fallot, interruption of the aortic arch, hypoplastic left heart syndrome, total anomalous pulmonary venous return, common arterial trunk, and pulmonary and tricuspid atresia. Heart murmur was the most common symptom in the group of acyanotic defects including ventricular and atrioventricular septal defects, coarctation of the aorta, and pulmonary and aortic stenosis; and in the group of cyanotic defects including double outlet right ventricle and Ebstein’s anomaly. Cyanosis was logically significantly more common in the group of cyanotic defects [cyanotic 69% (57/83) vs. acyanotic 8% (16/199); Pearson’s chi-square test, *p* < 0.0001, Odds ratio 25.1, 95% CI (12.1–53.3)]. Heart murmur was significantly more common in the group of acyanotic defects [cyanotic 24% (20/83) vs acyanotic 63% (125/199), Pearson’s chi-square test, *p* < 0.0001, Odds ratio 5.3, 95% CI 2.9–10.0].

**Table 3:** Symptoms of major heart defects leading to diagnosis

Congenital heart defect (***n*** = 282)	cyanosis N (%)	dyspnoea tachypnoea N (%)	failure to thrive N (%)	murmur N (%)	stigma- tization N (%)	weakened femoral arteries N (%)	circulatory instability N (%)	other organ disability N (%)
**Cyanotic defects**								
Transposition of the great arteries (*n* = 24)	24 (100)	0 (0)	0 (0)	0 (0)	0 (0)	0 (0)	0 (0)	0 (0)
Tetralogy of Fallot (*n* = 20)	13 (65)	0 (0)	0 (0)	7 (35)	0 (0)	0 (0)	0 (0)	0 (0)
Double outlet right ventricle (*n* = 14)	2 (14)	0 (0)	0 (0)	9 (65)	1 (7)	0 (0)	0 (0)	2 (14)
Common arterial trunk (*n* = 5)	4 (80)	1 (20)	0 (0)	0 (0)	0 (0)	0 (0)	0 (0)	0 (0)
Ebstein’s anomaly (*n* = 5)	1 (20)	0 (0)	0 (0)	4 (80)	0 (0)	0 (0)	0 (0)	0 (0)
Hypoplastic left heart syndrome (*n* = 4)	4 (100)	0 (0)	0 (0)	0 (0)	0 (0)	0 (0)	0 (0)	0 (0)
Pulmonary atresia/ventricular septal defect (*n* = 3)	3 (100)	0 (0)	0 (0)	0 (0)	0 (0)	0 (0)	0 (0)	0 (0)
Total anomalous pulmonary venous return (*n* = 3)	2 (67)	1 (33)	0 (0)	0 (0)	0 (0)	0 (0)	0 (0)	0 (0)
Interrupted aortic arch (*n* = 2)	2 (100)	0 (0)	0 (0)	0 (0)	0 (0)	0 (0)	0 (0)	0 (0)
Tricuspid atresia (*n* = 2)	2 (100)	0 (0)	0 (0)	0 (0)	0 (0)	0 (0)	0 (0)	0 (0)
Single ventricle (*n* = 1)	0 (0)	0 (0)	0 (0)	0 (0)	1 (100)	0 (0)	0 (0)	0 (0)
**Acyanotic defects**								
Ventricular septal defect (*n* = 72)	3 (4)	3 (4)	2 (3)	60 (83)	2 (3)	0 (0)	0 (0)	2 (3)
Persistent ductus arteriosus (*n* = 46)	0 (0)	0 (0)	2 (4)	21 (46)	0 (0)	0 (0)	23 (50)	0 (0)
Coarctation of the aorta (*n* = 33)	8 (24)	4 (12)	5 (15)	10 (31)	1 (3)	3 (9)	1 (3)	1 (3)
Atrioventricular septal defect (*n* = 19)	4 (21)	0 (0)	1 (5)	9 (48)	5 (26)	0 (0)	0 (0)	0 (0)
Pulmonary stenosis (*n* = 17)	1 (6)	1 (6)	0 (0)	15 (88)	0 (0)	0 (0)	0 (0)	0 (0)
Aortic stenosis (*n* = 12)	0 (0)	2 (17)	0 (0)	10 (83)	0 (0)	0 (0)	0 (0)	0 (0)

Circulatory instability and circulatory shock were most common in persistent ductus arteriosus cases due to immaturity and neonatological complications. Respiratory complications were the main symptom in 10–20% of the cases with coarctation of the aorta, aortic stenosis, total anomalous pulmonary venous return, and common arterial trunk. The highest rate of failure to thrive (15%) upon CHD diagnosis occurred in coarctation of the aorta. In this defect, finding of a weakened pulse on the femoral arteries contributed to diagnosis in only 9% of the cases. Stigmatisation due to genetic abnormalities contributed the most (26%) to the diagnosis of CHD in atrioventricular septal defect. Rarely, a diagnosis of CHD was made in the follow-up for other organ pathologies, most notably in cases of double-outlet right ventricle. Except for cyanosis and murmur, the incidence of symptoms between study groups differed in the failure to thrive [cyanotic 0% (0/83 vs. acyanotic 5% (10/199), Fisher’s exact test, *p* = 0.04] and circulatory instability [cyanotic 0% (0/83) vs acyanotic 12% (24/199), Fisher’s exact test, *p* = 0.0002], both of which were more common as a main symptom in the group of acyanotic lesions. For the remaining symptoms (i.e., dyspnoea, tachypnoea, weakened femoral arteries, and other organ disability), no difference was identified between the groups. Patients with symptoms other than cyanosis and murmur were more likely to be acyanotic [acyanotic 29% (58/199) vs. cyanotic 7% (6/83), Pearson’s chi-square test, *p* = 0.0001, Odds ratio 5.3; 95% CI: 2.1–15.6)].

## Discussion

CHDs are the most frequently observed morphological defects in human populations and most of them can be diagnosed prenatally. Although foetal echocardiography is a precise method for detecting cardiac malformations in the foetus and has excellent results, some defects are not detected prenatally and a child with an unrecognised heart defect can be born. The heart defect then manifests through different symptoms at different times. The main findings of this study were as follows: (i) despite the effectiveness of prenatal screening, half of the severe CHDs were undetected at birth; (ii) unrecognised heart defects had no perinatal major complications; (iii) three quarters of the children with prenatally unrecognised CHDs were symptomatic in the early neonatal period; and (iv) the most common symptoms of CHD were cyanosis and heart murmur.

The worldwide success rate of prenatal CHD diagnostics has generally improved [14], but the rates have varied among individual countries [15]. During this study, 57% of the severe CHDs (persistent ductus arteriosus excluded) were detected prenatally. Currently, the prenatal care system is very effective, and for some types of defects (single ventricle, hypoplastic left or right heart syndrome), the detection rate is 90–100%. Some families decided to terminate the pregnancy after receiving a prenatal CHD diagnosis, but prenatal diagnostics aim to carry out more detailed examinations of pathological pregnancies and correct counselling. Prior studies have provided different results regarding the beneficial effects of foetal diagnostics on the morbidity and mortality in newborns and children [14, 16–18]. However, in the authors’ opinion, foetal transport “in utero” for delivery at an adequate hospital is important. In these cases, the late diagnosis of a heart defect is not inevitable, and a child with CHD is stabilised and treated immediately after delivery.

Although the basic clinical signs of critical and severe heart defects are hypoxemia (cyanosis) and manifestations of heart failure, in our study newborns with unrecognised CHDs were without moderate or severe perinatal symptoms [9]. Newborns with CHDs had mostly normal Apgar scores after delivery, and symptoms of the defect developed later. Clinical stability was probably positively influenced by the presence of foetal shunts. Stable neonates are not routinely screened with a pulse oximeter on the first day in the study region, although this method would have good specificity and sensitivity to detect critical defects [19]. In addition, children with CHDs did not show a higher tendency towards premature birth or hypotrophy. The exception was children with a persistent ductus arteriosus; this diagnosis was due to their immaturity [20].

The CHD diagnosis was predominantly established in the early neonatal period (three quarters of the children), which is when the child is usually hospitalised in the neonatology department. Although clinical signs were dominant, we expect higher CHD detection under hospitalisation with the introduction of routine pulse oximetry [21]. In our study, all defects with future univentricular circulation requiring multiple operations were detected in the early neonatal period.

Some of the CHDs are not detected prenatally or postnatally in the hospital and the child is discharged home with an unidentified defect. Previous studies on delayed CHDs reported a rate of 20 to 30% neonates with CHDs undetected during birth hospitalisation [22, 23], which in our study was 25%. The number of these defects is influenced by the development of prenatal diagnostics. Another study showed 10% of delayed cases from all critical defects, which in our study corresponded to a 12% rate [24]. The late neonate stage is an important period for the newborn when foetal shunts close after discharge from the maternity hospital and severe or moderate CHD becomes apparent. The worst pre-operative condition and a critical course of the disease are more frequent in these newborns [25]. Two thirds of our delayed defects occurred in this late neonate period. According to the individual types of defects, coarctation of the aorta and Ebstein’s anomaly had the highest rate in this period. Ebstein’s anomaly is rare, but coarctation of the aorta has a significant incidence in the population. Up to 30% of coarctation cases are not diagnosed in newborns after delivery [24]; in our study it was 50%. Ventricular septal defect was most commonly detected in infants, although only severe and operated defects were included in this study. For this defect, surgery at high flow in symptomatic children early in infancy is recommended [26].

The most common symptoms in CHD were murmur and cyanosis. Heart murmur is common in healthy and asymptomatic children, but may also indicate a serious heart defect [27]. Newborns with heart murmur are at higher risk, as most asymptomatic newborns with heart murmur have a structural defect [28, 29]. In our study, ventricular septal defect and aortic and pulmonary stenosis were most often manifested by murmur. These defects are usually manifested by systolic murmur in asymptomatic patients [27]. Cyanosis is most often seen in the first weeks of life in children with CHD, when circulation is dependent on the arterial ductus [30]. In our patients, cyanosis was a major sign of critical defects that required repeated surgical corrections. Of well-operable defects, transposition of the great arteries typically manifested by cyanosis, mostly on the first day of life [31]. A murmur and cyanosis were the most important signs of CHD. In this study, newborns with murmur had 5 times higher chance of having acyanotic defects than those without murmur. Children with cyanosis, on the other hand, had (unsurprisingly) 25 times higher chance of having a cyanotic defect than children without cyanosis. Children with leading symptoms other than murmur and cyanosis were 5 times more likely to have acyanotic defects. This is important for more careful examination in children who do not have the most common symptoms of CHD.

Persistent ductus arteriosus constituted a special category as it is part of the fetal circulation and, therefore, represents a normal finding on foetal echocardiography. As postnatally, it can be a part of various complex defects, we have included it into the separate category only if present in an isolated form. This class was associated with the highest occurrence of circulatory instability. This, however, was influenced by the immaturity of neonates and neonatological complications. Still, it is, at present, not possible to accurately predict the development of circulatory instability in infants with persistent ductus arteriosus [32, 33]. In other CHDs, circulatory collapse was less common that in persistent ductus arteriosus, and the defects were detected according to clinical signs before severe alteration of circulation. This may indicate the quality of paediatric care in the monitored region. The clinical course of a child with CHD and beginning heart failure may imitate respiratory diseases, particularly dyspnoea and cough [34]. Other possible symptoms of CHD are poor feeding and failure to thrive. These were rare symptoms in our patients, and were most commonly found in children with aortic and aortic arch defects. Sometimes stigmatisation and suspected genetic abnormality indicated cardiological examination of the child. This was evident in atrioventricular septal defects, which were in a quarter of cases associated with a genetic impairment, especially trisomy 21 [35]. Surprisingly, coarctation of the aorta was rarely detected by examination of femoral artery pulsation in the study region. This examination is a screening test and the gold standard for the clinical detection of this defect [36]. This should improve with better education of paediatricians and a more focused attention during preventive examinations throughout childhood.

The main strength of the present study was the long-term follow-up in a region with a stable birth rate. The limitation of the study is the possible missing data on some cases – for example, the prenatally undetected defect may have not been registered in the event of the newborn’s death immediately after delivery in the regional hospital.

## Conclusion

In conclusion, murmur and cyanosis are the most common manifestations of prenatally undetected CHDs. Except for prenatally undiagnosed critical and severe defects, some cases with a persistent arterial ductus required operational solution, and cardiac symptoms were caused by persistent foetal circulation. Cyanotic lesions were diagnosed significantly earlier than defects with heart murmur. The majority of children with major heart defects were diagnosed as neonates. Diagnosis after discharge home occurred in 12% of all major CHDs.

## Supplementary Information

**Additional file 1: Table 1.** Newborns with prenatally detected or undetected CHDs, according to the genetic and morphological pathologies.

**Additional file 2: Table 2.** Association of CHDs and chromosomal aberrations.

## Data Availability

The datasets used and/or analysed during the current study are available from the corresponding author on reasonable request.

## Abbreviation

CHD: Congenital heart defects
