# Supplementary material for: Major heart defects: the diagnostic evaluations of first-year-olds
Source: BMC Pediatr. 2021 Nov 30;21:528. doi: 10.1186/s12887-021-02997-2 (PMC8630885; doi:10.1186/s12887-021-02997-2)
Supplement: Supplementary file 1 — Additional file 1: Table 1. Newborns with prenatally detected or undetected CHDs, according to the genetic and morphological pathologies. [file 12887_2021_2997_MOESM1_ESM.docx]

Table S1. Newborns with prenatally detected or undetected CHDs, according to genetic and morphological pathologies

| **CHD** | **Prenatally detected (%)** | | | | **Prenatally undetected (%)** | | | | **Total** |
| --- | --- | --- | --- | --- | --- | --- | --- | --- | --- |
|  | **n** | **CHD**  **I** | **CHD G** | **CHD M** | **n** | **CHD**  **I** | **CHD G** | **CHD M** |  |
| Aortic stenosis | **20** | 20 (100) | 0 (0) | 0 (0) | **12** | 10 (83) | 2 (17) | 0 (0) | **32** |
| Atrioventricular septal defect | **46** | 35 (76) | 9 (20) | 2 (4) | **19** | 13 (68) | 6 (32) | 0 (0) | **65** |
| Coarctation of the aorta | **24** | 20 (83) | 0 (0) | 4 (17) | **33** | 33 (100) | 0 (0) | 0 (0) | **57** |
| Common arterial trunk | **6** | 6 (100) | 0 (0) | 0 (0) | **5** | 5 (100) | 0 (0) | 0 (0) | **11** |
| Corrected transposition of the great arteries | **3** | 3 (100) | 0 (0) | 0 (0) | **0** | 0 (0) | 0 (0) | 0 (0) | **3** |
| Double outlet right ventricle | **23** | 22 (96) | 1 (4) | 0 (0) | **14** | 13 (93) | 0 (0) | 1 (7) | **37** |
| Ebstein’s anomaly | **6** | 6 (100) | 0 (0) | 0 (0) | **5** | 5 (100) | 0 (0) | 0 (0) | **11** |
| Hypoplastic left heart syndrome | **41** | 40 (98) | 0 (0) | 1 (2) | **4** | 4 (100) | 0 (0) | 0 (0) | **45** |
| Interrupted aortic arch | **2** | 2 (100) | 0 (0) | 0 (0) | **2** | 2 (100) | 0 (0) | 0 (0) | **4** |
| Persistent ductus arteriosus/isolated | **-** | - | - | - | **46** | 45 (98) | 1 (2) | 0 (0) | **46** |
| Pulmonary atresia/intact ventricular septum | **6** | 6 (100) | 0 (0) | 0 (0) | **0** | 0 (0) | 0 (0) | 0 (0) | **6** |
| Pulmonary atresia/ventricular septal defect | **9** | 9 (100) | 0(0) | 0 (0) | **3** | 3 (100) | 0 (0) | 0 (0) | **12** |
| Pulmonary stenosis | **22** | 21 (95) | 1 (5) | 0 (0) | **17** | 16 (94) | 1 (6) | 0 (0) | **39** |
| Single ventricle | **7** | 4 (57) | 0 (0) | 3 (43) | **1** | 0 (0) | 0 (0) | 1 (100) | **8** |
| Tetralogy of Fallot | **34** | 26 (76) | 7 (21) | 1 (3) | **20** | 20 (100) | 0 (0) | 0 (0) | **54** |
| Total anomalous pulmonary venous return | **0** | 0 (0) | 0 (0) | 0 (0) | **3** | 3 (100) | 0 (0) | 0 (0) | **3** |
| Transposition of the great arteries | **31** | 29 (94) | 0 (0) | 2 (6) | **24** | 24 (100) | 0 (0) | 0 (0) | **55** |
| Tricuspid atresia | **8** | 8 (100) | 0 (0) | 0 (0) | **2** | 2 (100) | 0 (0) | 0 (0) | **10** |
| Ventricular septal defect | **28** | 23 (82) | 4 (14) | 1 (4) | **72** | 63 (87) | 5 (7) | 4 (7) | **100** |
| Total | **316** | **280 (89)** | **22 (7)** | **14 (4)** | **282** | **261 (92)** | **15 (6)** | **6 (2)** | **598** |

CHD-congenital heart defect, I-isolated, G-concomitant genetic disorder, M-extracardiac malformation with a normal karyotype
