# Supplementary material for: Major heart defects: the diagnostic evaluations of first-year-olds
Source: BMC Pediatr. 2021 Nov 30;21:528. doi: 10.1186/s12887-021-02997-2 (PMC8630885; doi:10.1186/s12887-021-02997-2)
Supplement: Supplementary file 2 — Additional file 2: Table 2. Association of CHDs and chromosomal aberrations. [file 12887_2021_2997_MOESM2_ESM.docx]

**Table S2. Association of CHDs and chromosomal aberrations**

| **CHD (n=598)** | **Chromosomal aberrations (%)** |
| --- | --- |
| Aortic stenosis (n=32) | 2 (6%)  1x Deletion 7q11.23, 1x Noonan syndrome |
| Atrioventricular septal defect (n=65) | 15 (23%)  12x Trisomy 21, 2x Trisomy 18, 1x Smith-Magenis syndrome |
| Coarctation of aorta (n=57) | 0 (0%) |
| Common arterial trunk (n=11) | 0 (0%) |
| Corrected transposition of great arteries (n=3) | 0 (0%) |
| Double outlet right ventricle (n=37) | 1 (3%)  1x Partial deletion 11q |
| Ebstein’s anomaly (n=11) | 0 (0%) |
| Hypoplastic left heart syndrome (n=45) | 0 (0%) |
| Interrupted aortic arch (n=4) | 0 (0%) |
| Persistent ductus arteriosus/isolated (n=46) | 1 (2%)  1x Trisomy 21 |
| Pulmonary atresia/intact ventricular septum (n=6) | 0 (0%) |
| Pulmonary atresia/ventricular septal defect (n=12) | 0 (0%) |
| Pulmonary stenosis (n=39) | 2 (5%)  1x monosomy X/inv. 4, 1x Deletion 7q11.23 |
| Single ventricle (n=8) | 0 (0%) |
| Tetralogy od Fallot (n=54) | 7 (13%)  4x Deletion 22q11, 3x Trisomy 21 |
| Total anomaly of pulmonary venous return (n=3) | 0 (0%) |
| Transposition of great arteries (n=55) | 0 (0%) |
| Tricuspid atresia (n=10) | 0 (0%) |
| Ventricular septal defect (n=100) | 9 (9%)  6x Trisomy 21, 1x Deletion 22q11, 1x Trisomy 18,  1x Smith Magenis syndrome |
| Total | 37 |

CHD-congenital heart defect
